# Vulnerable mothers’ experience of feeding their preterm infant in neonatal care

**DOI:** 10.4102/sajcd.v68i1.842

**Published:** 2021-10-28

**Authors:** Elanie A. van Schalkwyk, Berna Gerber

**Affiliations:** 1 Department of Speech, Language and Hearing Therapy, Faculty of Medicine and Health, Stellenbosch University, Cape Town, South Africa

**Keywords:** vulnerable mothers, experiences, feeding, breastfeeding, preterm infants, neonatal care

## Abstract

**Background:**

Worldwide, preterm birth is a rising threat to maternal and child health. The universal challenges of being the mother of a preterm infant, combined with context-specific challenges such as poverty and poor linguistic and cultural representation, bring about risks for both mother and infant. This includes poor maternal mental health; poor mother–infant bonding and attachment and potential suboptimal developmental outcomes for the infant.

**Objective:**

This article describes how Afrikaans-speaking mothers living in poverty experienced feeding their preterm infants in neonatal care.

**Method:**

The study implemented a cross-sectional, qualitative design. Mothers of preterm infants (chronological ages between 3 and 6 months) were selected through a purposive sampling method and participated in individual semi-structured interviews. Nine interviews were thematically analysed. The participants were a vulnerable group, about whom little research literature was available.

**Results:**

Feeding was perceived as a progressive task that worked towards the goal of discharge from the hospital. It was stressful because of various factors, but insufficient breastmilk supply was a significant contributor. The hospital setting was perceived as something that added to the participants’ anxiety surrounding feeding, but at the same time, it had the potential to decrease their anxiety. When the mother–infant dyad was able to breastfeed successfully, it made the participants feel like mothers at last after an extended period of anticipation.

**Conclusion:**

Feeding their preterm infant was a prominent experience for the mothers, especially whilst in neonatal care. Increased feeding support is required from the healthcare team providing neonatal care in order to optimally use the neonatal period.

## Introduction

An increasing number of infants in South Africa are at risk of neurodevelopmental delays because of the high frequency of preterm birth (South African Speech-Language-Hearing Association [SASLHA], 2017). South Africa presents various risk factors for preterm birth that also complicate caring for a preterm infant. These factors include the high burden of disease, resource constraints in various respects and on many levels and inequalities relating to social determinants of health (Dawes, Biersteker, & Irvine, 2008). As modern technology and advances in neonatal care are ensuring the survival of preterm infants at younger gestational ages and with lower birthweights (World Health Organisation [WHO], 2017a), low- and middle-income countries are experiencing increased neonatal morbidity rates and challenges to already overburdened and poorly resourced health services (Pusdekar et al., 2020).

Being the mother of a preterm infant is universally described as a challenging and stressful experience. Preterm infants are at risk of various medical, neurological and developmental complications that may influence current and future feeding skills (Crapnell et al., 2013). As a result of neurological, sensory and physiological immaturity, preterm infants often display neurobehavioral dysfunction and consequently, the achievement of skills fundamental for successful oral feeding is delayed or disordered (Brown et al., 2009). These skills include: state regulation, motor organisation, rhythmical sucking and coordinating a suck-swallow-breath pattern (Crapnell et al., 2013). The potential comorbidities of preterm babies may have physical, psychological, social and financial implications for their mothers in the short and/or long term (Petrou, 2005). The mother of a preterm infant has a unique early parenting experience in caring for her infant (Pascoe, Bissessur, & Mayers, 2016), which may potentially alter the nature of mother–infant interactions in the early months of life.

Mothers of preterm infants with low socio-economic status (SES) and from linguistic minority groups face additional challenges that make them especially vulnerable and may negatively influence their experience of caring for their preterm infant. In addition to the trials associated with poverty, they may have limited linguistic and cultural representation within the health system. In South Africa, mothers with a low SES from all of the 11 indigenous South African languages, including Afrikaans, are in such a situation. The focus of the current study was on poor Afrikaans-speaking mothers of preterm infants. Firstly, living in poverty means that they experience high levels of psychosocial stress in combination with limited access to social and economic resources (Crapnell et al., 2013). Secondly, despite Afrikaans being one of South Africa’s 11 indigenous languages, Afrikaans mothers constitute a group that is poorly represented in a health system where English is the language of choice of many healthcare professionals (HCPs) (Penn & Watermeyer, 2018). The universal challenges of being a mother of a preterm infant, combined with the context-specific challenges of belonging to an underserved and socially marginalised group, bring about risks for both mother and infant. This includes: poor maternal mental health, poor mother–infant bonding and attachment and potential sub-optimal feeding and communication development.

Existing early communication intervention (ECI) guidelines emphasise the involvement of the mother (primary caregiver) in the intervention process (Craig et al., 2015) as mothers’ perceptions and experiences of caring for their infant have a strong influence on the future communication and feeding development of the child. The ECI services provided to vulnerable populations, such as Afrikaans mothers of preterm infants with low SES, should not only be sensitive but also accommodate their unique perceptions and experiences. Such an approach will allow speech-language therapists to utilise the neonatal period optimally, to support mothers in stimulating communication and feeding development and to prevent or timeously identify communication and feeding delays or disorders (SASLHA, 2017). Not much research, either local or international, has been published on the experiences of mothers from linguistic minority groups who live in poverty in caring for a preterm infant. The poor representation of these vulnerable groups in the research literature and the consequent inadequate professional knowledge render them susceptible to healthcare that is neither contextually appropriate nor sensitive towards their unique realities.

The aim of the study was to describe and explain how Afrikaans mothers living in low socio-economic circumstances in the Western Cape province experience feeding their preterm infant in neonatal care. The goal was achieved by articulating the following research question: *How do Afrikaans mothers, living in low socio-economic circumstances in the Western Cape province, experience feeding their preterm infant in neonatal care?* The answer to this question may allow South African speech-language therapists to determine more accurately ‘what works best for whom, when and how’ (SASLHA, 2017, p. 3) with regard to ECI for at-risk neonates and mothers in our culturally and linguistically diverse context.

## Method

### Study design

This study entailed a cross-sectional, qualitative design. A small group of participants were selected through a purposive sampling method, more specifically criterion sampling. They participated in individual in-depth interviews that were guided by a semi-structured discussion schedule. This study design was appropriate as it assisted in exploring and describing the ‘meaning for several individuals of their lived experiences of a concept or phenomenon’ (Creswell & Poth, 2016, p. 57). The initial phenomenon in question was maternal experiences of caring for a preterm infant in the first months of life. Following data collection, the data-led decision was made to provide an in-depth account of a more specific phenomenon, namely maternal experiences of feeding their preterm infant whilst in neonatal care. This decision will be expanded upon in the data collection and analysis sections.

### Study sampling strategy

The sample frame entailed all Afrikaans mothers of preterm infants with ages ranging from 3 to 6 months (chronological age) who attended follow-up appointments at a High-Risk Clinic at a Paediatrics out-patient department of a public tertiary hospital. Purposive sampling, more specifically criterion sampling, was used to select participants. The inclusion criteria stipulated that participants must be Afrikaans first-language speakers, older than 18 years, have low SES and be the mother of a preterm infant. Infant-specific criteria included that the infant must be born before 37 weeks (Quinn et al., 2016), born with birthweight of less than 2500 g (Quinn et al., 2016), aged 3–6 months (chronological age) at the time of data collection, medically stable at the time of data collection and with no diagnosed biological, physical, sensory, congenital, or neurological disorders at the time of data collection. Low SES was determined by limited educational attainment (no post-matriculation degree of diploma) and financial income (bracket H0 – H1 on the patient income scale that represents household with a total annual income of R0 – R100 000). Prematurity was classified as extremely preterm (< 28 weeks), very preterm (28 – < 32 weeks) and moderate or late preterm (32 – < 37 weeks) (Quinn et al., 2016). Birthweight was classified as low birthweight (< 2500 g), very low birthweight (< 1500 g) and extremely low birthweight (< 1000 g) (Quinn et al., 2016).

In addition to the inclusion criteria, specified characteristics pertaining to the participants and their infants were considered in order to ensure variation within the sample (as listed in Table 1). This variation aimed to increase the transferability of the study. The characteristics included: the participant’s age, number of children, highest education level attained, employment status and relationship status. The infant’s degree of prematurity and birthweight were further considered. Variation in the sample ensured that the participants were heterogeneous and provided diverse experiences of the phenomenon being explored (Malterud, Siersma, & Guassora, 2016).

**TABLE 1:** Characteristics of the nine study participants.

Participants	Age (years)	Education	Employment	Relationship status	Children (total)	Infant gestational age (weeks)	Infant birthweight (g)
1	32	Grade 11	Cleaner	Married to infant’s biological father	3	26	<2500
2	32	Grade 10	Quality controller	Relationship with infant’s biological father	1	30	<2500
3	18	Grade 10	Unemployed	Relationship with infant’s biological father	1	28	1300
4	24	Grade 12	Unemployed	Relationship with infant’s biological father	1	27	940
5	22	Grade 12	Unemployed	Married to infant’s biological father	1	32	1360
6	36	Grade 10	Admin clerk	Single	2	29	1000
7	31	Grade 10	Unemployed	Married to infant’s biological father	4	29	1250
8	22	Grade 12	Carer at retirement home	Relationship with infant’s non-biological father	1	29	955
9	38	Grade 7	Farmworker	Married to infant’s biological father	5	33	1290

Mothers who met the inclusion criteria were identified by researcher one and the paediatrician responsible for the clinic and invited to participate in the study following their paediatric appointment. This process continued until data saturation was reached after 11 interviews conducted over 5 days. The selected sample was reviewed by the researcher for a second time to confirm that all the inclusion criteria were met and to ensure that the interviews were of sufficient quality for inclusion in the study. The final sample consisted of nine (n = 9) participants. The heterogeneous nature of the selected sample (as listed in Table 1), the detail-rich narratives collected during the interviews, and the decision to use in-depth analysis strategies to provide a detailed account of this phenomenon, assisted in establishing when data saturation occurred (Malterud et al., 2016).

### Study context (population and setting)

The study population entailed Afrikaans-speaking mothers of preterm infants with low SES. This may be viewed as a vulnerable group that is at risk for poor healthcare outcomes with regard to three attributes (Clark & Preto, 2018), namely being the mother of a preterm infant (health attribute), low SES (economic attribute) and belonging to a linguistic minority group (cultural attribute). All participants attended a medical follow-up appointment at a High-Risk Clinic with their infant at the same public tertiary hospital. During their infant’s admission, all mothers lodged in with their infants and the infants received neonatal care from the same public tertiary hospital. Mothers are thus considered to have had similar in-patient care, follow-up care, and support provided from HCPs. The hospital adhered to the Baby-Friendly Hospital Initiative as outlined by the WHO and the United Nations International Children’s Emergency Fund (WHO, 2017b). This initiative promotes the provision of breastfeeding support to mothers, the use of expressed breastmilk whilst the infant is unable to breastfeed (as opposed to formula milk), exclusive breastfeeding (as opposed to bottle feeding); and rooming in of mothers and infants (WHO, 2017b ). Whilst in neonatal care, mothers were provided with galactagogues as the need arose. The healthcare team typically included medical doctors, nursing staff and dieticians. All mothers in neonatal care did not receive routine feeding and/or swallowing intervention and support from speech-language therapists as these services were provided on a referral basis for infants with identified feeding difficulties. Feeding support (whilst tube feeding, cup feeding and breastfeeding) was mostly the responsibility of the nursing staff. Breast pumps were not available and mothers had to hand express breastmilk, except if they had personal access to a breast pump. The hospital is a large facility that employs HCPs from various cultural and linguistic backgrounds although services are mostly delivered in English. Culture and language differences were thus likely to exist between some of the participants and the HCPs.

### Data collection

Eleven interviews were conducted in a private consulting room near the clinic using a semi-structured discussion schedule. Interviews were conducted 1–3 months after the mother–infant dyad was discharged from the hospital. An initial pilot study was completed with three participants to ensure the credibility of the discussion schedule and the interview process. Following review and discussion of the pilot study between the authors and an external expert in the field, no changes were made to the discussion schedule and minimal changes were recommended for the interview process. Two of the interviews from the pilot study were deemed suitable and formed part of the study data. A total of nine interviews were included in the study.

The discussion schedule (see Appendix 1) entailed a revised version of a semi-structured questionnaire compiled by Buys (2020) who completed a similar study amongst isiXhosa speaking mothers. Buys’s (2020) questionnaire was translated into Afrikaans, and the content was reviewed by the authors to facilitate an open-ended discussion. The discussion schedule explored seven key areas related to caring for a preterm infant, namely maternal perceptions regarding prematurity, caregiving, feeding, mother–infant interaction, maternal responsibilities, additional role-players in their infant’s care and development and information needs. The open-ended nature of the discussion schedule allowed participants to guide the discussion and aimed to increase the confirmability of the tool (Ali & Yusof, 2012). The participants spontaneously gravitated towards the topic of feeding their preterm infant whilst hospitalised. It was clear that feeding their preterm infant in neonatal care was a prominent experience and one that participants could recall vividly. The interviews were conducted in Afrikaans, had a duration of approximately 45 min and were voice recorded. Author one conducted the interviews and is an Afrikaans first-language speaker. The voice recordings were transcribed verbatim by author one. To ensure dependability and confirmability of the transcripts, 22% of the data were externally audited by a Speech-Language Pathology doctoral student. To ensure confirmability of the translation of the participants’ quotations from Afrikaans to English, it was reviewed using a forward and back translation procedure involving author one and an external language editor.

### Data analysis

Thematic analysis, as described by Braun and Clarke (2006), was used to analyse the data. Analysis was aimed at providing a detailed account of one particular aspect (maternal experiences of feeding their preterm infant whilst in neonatal care) as opposed to an overview of the entire data set (maternal experiences of caring for their preterm infant in the early months of life). This decision aimed to increase the study’s credibility and confirmability as it became clear during data collection that certain aspects (feeding) and periods (neonatal care) were prominent experiences to the mothers of preterm infants. An inductive approach to thematic analysis was used by creating semantic codes and themes that focused on the explicit meaning of the collected data. Author one familiarised herself with the data by conducting the interviews, transcribing the audio recordings, reading the transcripts repeatedly and documenting early analytical remarks. The transcripts were coded in an iterative fashion, and data extracts were grouped into six main themes. The computer-assisted qualitative data analysis software (CAQDAS) programme *ATLAS.ti 8 Windows* was used to assist with managing and organising codes and themes. Refined codes and themes were assigned following various reviews and discussions between the authors to increase the credibility of the study (Ali & Yusof, 2012).

## Findings

Six themes (outlined in Table 2) arose during data analysis and provide a detailed account of the participants’ experiences of feeding their preterm infants whilst in neonatal care. Participants provided vivid descriptions of their experiences of feeding their preterm infants, especially concerning the period during which the infant was hospitalised. The length of an infant’s hospital stay differed according to infant-specific factors such as medical stability, gestational age and birthweight. The time infants spent in hospital ranged from 3 to 11 weeks. The term ‘feeding’ includes all aspects related to the task such as mothers having to express breastmilk, the environment in which the feeding task occurred, the various feeding methods the infant progressed through and the infant’s ability to participate in feeding tasks (sucking and swallowing skills).

**TABLE 2:** Themes describing maternal experiences of feeding a preterm infant whilst hospitalised.

Number	Theme
1	Feeding was a progressive task aimed at ‘going home’
2	Feeding was a significant contributor to stress ‘in the beginning’
3	Breastmilk was ‘the biggest thing’ in hospital
4	Breastfeeding was ‘amazing’
5	The hospital setting was both stressful and helpful
6	Feeding became ‘easier’ as mother and infant ‘got used to it’

### Feeding was a progressive task aimed at ‘going home’

‘It was very stressful. Very, very, very. Because I knew the breast is what’s going to let her go home. And her weight was already 1.8 [*kilograms*] and then she still couldn’t go [*home*] because she couldn’t breast [*breastfeed*].’ (Participant 1, Grade 11, Cleaner)

‘To me it just felt like I was never going to get there [*referring to 1.8 kilograms*] and it’s still far because they said he must be this certain weight before we can move [*go home*].’ (Participant 8, Grade 12, Carer at retirement home)

‘In the beginning, the infant is not with you. He is in the incubator. For now, it must be like this because he is still too small. And then you start to feed, they [*nursing staff*] teach you how to feed the baby. Cup feeding and after cup feeding, you go to another ward where your baby is with you. And from there you go home.’ (Participant 9, Grade 7, Farmworker)

Feeding the infant was experienced as a constantly changing task that was closely tied to the goal of discharge. Mothers described the following as prerequisites for attaining this goal: successful breastfeeding, and steady weight gain with a target weight of 1.8 kg or more. The mothers’ accounts reflected the feeding-related discharge criteria of the hospital namely that the infant should be able to take adequate oral feeds (preferably via breastfeeding) with no safety concerns such as cyanosis, apnoea or vomiting and demonstrate an upward weight gain trend. This may be an indication that these criteria were communicated clearly to the participants. The task itself was perceived to change with regard to two aspects, namely the method of feeding and the feeding volume. Firstly, the method of feeding the infant entailed ‘something new’ on a frequent basis. Mothers described their infant’s feeding journey as starting with tube feeding (orogastric or nasogastric tubes) whilst the infant was in the incubator. Following this, infants progressed to cup feeding and occasionally syringe feeding. Lastly, the mother–infant dyad was challenged to progress to breastfeeding. Breastfeeding was associated with moving out of the high care ward and perceived as a big step towards discharge. These changes contributed to maternal stress, as the mother was repeatedly expected to rebuild her feeding skill and confidence using a new method. Secondly, the feeding volumes always needed to be ‘more’. The millilitres of milk the infant was supposed to consume during a feed were perceived to go ‘higher and higher every day’. Thirdly, the infant was expected to gain weight steadily and this was monitored and documented daily. This monitoring process often resulted in feelings of hopelessness, and the infant’s weight was perceived as a numerical indicator of the infant’s progress towards the eventual goal of discharge.

### Feeding was a significant contributor to stress ‘in the beginning’

‘In the beginning when he was born, I struggled with the milk and that stressed me out a bit because they [*HCPs*] told me that he must get my milk because it will help him quicker.’ (Participant 8, Grade 12, Carer at retirement home)

‘So to me it was okay in the beginning, it was just sad that I couldn’t breastfeed him. I cried a lot because I couldn’t breastfeed him because I thought my child is never going to take the breast.’ (Participant 6, Grade 10, Admin clerk)

The task of feeding their infant was a significant cause of stress for the participants and was especially prominent ‘in the beginning’ of the infant’s life. Various factors related to feeding were described as contributors to the mothers’ anxiety, including concerns regarding poor weight gain and growth of the infant, messing during feeding times, insufficient breastmilk supply and difficulty with the task of breastfeeding itself. The progressive nature of the feeding task contributed significantly to maternal anxiety. Mothers were striving towards the goal of discharge from the hospital and therefore any factor that interfered with the attainment of this goal added to maternal stress.

### Breastmilk was ‘the biggest thing’ in hospital

‘That was such a big stress to me, the milk. And then they move it up, they move it up up up – at the end, it is so much milk that I can’t even almost give that total.’ (Participant 7, Grade 10, Unemployed)

‘And they were just like you must express milk and I was like I can’t. Even that where you must wake up every two hours to express, I had to do that even though I didn’t have milk and nothing comes!’ (Participant 4, Grade 12, Unemployed)

The mothers experienced their breastmilk supply as one of the biggest problems during the hospital stay. They preferred their infants to receive ‘my milk’ as they believed this would help their infant the ‘quickest’ by facilitating steady weight gain and, subsequently, an expedited discharge. Firstly, mothers were concerned that they would not be able to meet the prescribed millilitres as the infant’s feeding volumes increased on a frequent basis. Secondly, the process of expressing milk was hard for mothers as it was often novel, painful or fruitless. Thirdly, mothers felt overwhelmingly bombarded with the message of expressing breastmilk and perceived nursing staff to ‘push’ and ‘force’ them to express their milk even though it was clear that they were struggling with lactation. It appears that breastmilk was perceived as an elixir in hospital. Having a good supply made you rich whilst a limited supply left you poor and hopeless. The mothers of extremely or very preterm infants did feel hopeless during most of their hospital stay.

### Breastfeeding was ‘amazing’

‘The Saturday when the sister told me I must breastfeed him it was such an amazing feeling that he was on my breasts… I can’t explain it to anyone that amazing feeling, it’s like butterflies that I felt inside of me when he drank from me the first time.’ (Participant 4, Grade 12, Unemployed)

‘It made me feel good because now it feels to me like okay, now I am a mother at last (laughs). I am now a mother because he drinks from me.’ (Participant 8, Grade 12, Carer at retirement home)

Mothers experienced breastfeeding their infant as an exceptionally positive experience which they thoroughly enjoyed. Breastfeeding was described as advantageous to both mother and child. Physiological advantages described by the mothers include increased breastmilk production, weight gain for the infant, a healthy infant, and lastly discharge from hospital. Interaction-attachment advantages described include increased bonding, proximity and communication between the mother and the infant. After having had to rely on alternative methods to feed their infants, such as orogastric and nasogastric tube feeding, syringe feeding, and cup feeding, breastfeeding made the mothers feel like mothers for the first time.

### The hospital setting was both stressful and helpful

‘It was actually harder in hospital than it was at home… I think it’s because the hospital is very controlled like you must feed at this time, you must change the nappy when you feed her, you must do it… but at home, it’s more like they give signals when the nappy is wet or… like she will cry when she’s hungry.’ (Participant 4, Grade 12, Unemployed)

‘If you must stay, you must stay – it is your child’s health. You can’t be against it.’ (Participant 9, Grade 7, Farmworker)

‘It’s just sometimes the nurses were a little… when we feed. They were actually strict.’ (Participant 1, Grade 11, Cleaner)

The hospital setting was perceived as novel, controlled, and anxiety-provoking although mothers were willing to endure this context to ensure their infants received the intervention they required. The support offered by the HCPs on how to complete caregiving tasks was valuable and appreciated although nursing staff were occasionally perceived to be impatient and strict.

### Feeding became ‘easier’ as mother and infant ‘got used to it’

‘I learned everything here. I didn’t know anything about premature. I learned everything day after day… observed the nurses, listened to what the doctors are talking, and then I also started watching her and this is how I learned.’ (Participant 1, Grade 11, Cleaner)

‘Their insides are almost like not fully developed… like where a full nine-month baby when you give birth, they give your baby to you and you put baby on the breast and the baby clicks immediately I must drink now and so on. The difference is with them that they can’t just drink when they come out, they are like they don’t know that they must drink.’ (Participant 7, Grade 10, Unemployed)

‘She messed for the first few days – spit it [*milk*] out, didn’t really know how to swallow it. And then afterwards it got easier for me.’ (Participant 5, Grade 12, Unemployed)

The mothers felt that they became more comfortable with feeding their infants as they acquired the following skills: recognising the infant’s hunger cues, identifying the infant’s specific preferences, handling the infant and using feeding equipment. They described three methods whereby they learned these skills, namely observation, direct instructions from the HCPs and self-guidance. With regard to their infants’ learning, they experienced that ‘in the beginning’ their infants didn’t immediately ‘click’ that they must suck and did not know how to suck because they were not as fully developed as a full-term infant. According to the mothers, their infants ‘got used to it’ with exposure and practice, and learned how to feed.

## Discussion

The participants experienced the task of feeding their infant as a significant stressor related to caring for their infants, especially whilst the infant was hospitalised. This finding frequently appears in the research literature (Buys, 2020; Ericson & Palmér, 2019; Flacking, Ewald, Nyqvist, & Starrin, 2006; Leonard & Mayers, 2008; Swift & Scholten, 2009), and various factors are thought to contribute to feeding being a challenging task (Van Schalkwyk, Gay, Miller, Matthee, & Gerber, 2020). The most prominent contributor in this study was the strong association between successful oral feeding and discharge from the hospital. Participants described feeding as a progressive task that worked towards one goal, namely ‘going home’. The mothers formulated a clear recipe for discharge from the hospital: present a breastfeeding infant with a weight of at least 1.8 kg. This strong association between successful oral feeding and discharge from the hospital is perhaps a major cause of the anxiety surrounding feeding. Successful feeding results in discharge from the hospital, whilst difficulty with feeding results in an extended hospital stay.

An important question to ask is why the mothers associated successful oral feeding so closely with discharge from the hospital. According to Lubbe (2018), it is because of the volume-driven approach to feeding as well as the intense monitoring of oral feeding within the neonatal hospital context (Thoyre, 2001). The participants in the current study felt bombarded with the message that they must express breastmilk and supply specified amounts of breastmilk for their infant as breastmilk is ‘the thing that is going to send us home the quickest’. The big focus and daily monitoring of the infant’s weight gain gave the participants a perception of the infant’s weight as a numerical measure of their progress towards discharge and had a strong influence on maternal emotions.

The way that HCPs package and convey messages regarding feeding to the mothers of preterm infants should be carefully considered. Firstly, HCPs need to adjust the packaging of their message to accommodate the hardships that vulnerable mothers of preterm infants encounter. The following hardships were highlighted in the current study and are also supported by literature: high stress levels (Van Schalkwyk et al., 2020), difficulty with initiating and maintaining lactation (Ericson & Palmér, 2019; Lee & Gould, 2009), reduced feeding abilities of the infant (Crapnell et al., 2013), limited reciprocal interaction with their infant (Pascoe et al., 2016) and being in an unfamiliar hospital environment for prolonged periods with limited support (Swift & Scholten, 2009). An important recommendation for health professions education is thus to focus on developing awareness of and sensitivity towards the hardships vulnerable mother–infant dyads from diverse backgrounds experience and skills in addressing them (Penn, 2014).

Secondly, HCPs should find creative ways to motivate mothers to use expressed breastmilk and to breastfeed their infants as prescribed by the WHO (2020). Perhaps it is necessary to ask whether breastmilk is being promoted by focusing on all the advantages it entails for mother and infant or whether it is simply promoted as ‘the thing that is going to send us home the quickest’ and that may result in an expedited discharge. Promoting the use of breastmilk and breastfeeding by focusing on the inherent advantages versus focusing on an expedited discharge may produce different long-term outcomes in adhering to these practices. The first option may increase internal motivation to follow the prescribed health behaviours. The second option runs the risk of producing an intense and likely unsustainable short-term motivation to use breastmilk and breastfeed as a means to an end (discharge from hospital). It is recommended that future research efforts explore ways in which the HCPs can encourage mothers of preterm infants to use breastmilk or to breastfeed, in a way that is sensitive towards their realities, and ensures their long-term commitment to these practices. This recommendation becomes especially relevant if one considers that all study participants converted to bottle and formula feeding once they returned home. Some of the participants explained that this shift was because of their return to work, whilst others stated that this was either the infant’s or their own preference. This finding corresponds with the findings of Buys (2020) who conducted a similar study amongst isiXhosa speaking mothers.

The findings of this study have clinical implications for the way in which HCPs involved in neonatal care are currently providing feeding support to mothers of preterm infants. Firstly, the situation requires increased awareness and acknowledgement of the challenges the mothers of preterm infants from low socio-economic settings face. Secondly, HCPs should increase their focus on mothers as equally important receivers of intervention whilst the mother–infant dyad is hospitalised. Thirdly, creative ways should be considered to provide emotional support and protect the emotional wellbeing of these mothers whilst their infants are hospitalised. Potential support methods include in-hospital support groups, a system to flag and monitor mothers at risk of mental illness and access to mental health services if indicated. Fourthly, bonding and the attainment of a maternal identity should be encouraged during all stages of the preterm infant’s hospital stay. This can be achieved by actively including mothers in all caregiving tasks, especially feeding, from the start of the infant’s journey. This can further be achieved by encouraging Kangaroo Mother Care (KMC), as skin-to-skin contact is an evidence-based nursing intervention that offers benefits to preterm infants, their mothers and also the health system (Pattinson, Bergh, Malan, & Prinsloo, 2006). These benefits include the promotion of bonding, maternal sensitivity towards the infant’s cues, lactation and breastfeeding (Kritzinger & Van Rooyen, 2014). Lastly, whilst in neonatal care, HCPs have the opportunity and obligation to counsel mothers regarding prematurity to ensure they comprehend their infant’s behaviour and needs during the maturation process. This should include discussions surrounding developmental stages, stress signs, communication cues, appropriate sensory stimulation and feeding support (Pike, Kritzinger, & Krüger, 2017).

A further necessary clinical recommendation is for the mothers of preterm infants to have increased access to feeding and swallowing intervention from speech-language therapists. Preterm infants are known to have delays and/or difficulties in the establishment of oral feeding (Crapnell et al., 2013), and speech-language therapists may provide effective intervention to the mother–infant dyad to facilitate the gradual progression to safe and adequate oral feeding. Additionally, access to lactation consultants might be of further assistance to mothers as the findings highlighted that initiating and maintaining lactation was a significant challenge to the participants.

It is important to mention that linguistic and cultural differences between the participants and the HCPs were not frequently mentioned or vividly described by the participants. One participant explained that whilst she was in hospital, she was dependent on her husband or other mothers in the ward to convey HCPs’ messages regarding the infant to her as she has limited comprehension of complex English conversations. It can be deduced that the participant had two options with regard to receiving counselling about her preterm infant’s condition, namely to wait until her husband was present (delaying receiving much anticipated information); or to ask a stranger to assist (potentially sharing sensitive and private information). This was the only instance where a participant mentioned language or culture as a barrier to receiving information and health care, albeit a powerful example of receiving ‘monolingual health services in a multilingual society’ (Elkington & Talbon, 2016). Linguistic and cultural differences may be more prominent between HCPs and participants who speak other indigenous languages (besides Afrikaans).

### Limitations of the study

Firstly, the infants’ birthweights were specified as ‘under 2500 g’ in the pilot study, whilst the exact birthweight was documented in the main study. The researcher is therefore able to provide only a limited description (gestational age only) of the preterm infants included in the pilot study and a more detailed description (gestational age and birth weight) of the preterm infants is included in the main study. Secondly, the SES of the participants was determined by their financial income and educational attainment. Information about participants’ housing was not collected and may have provided additional insight into the mothers’ economic situation.

## Conclusion

Mothers of preterm infants with low SES and from linguistic minority groups, such as poor Afrikaans-speaking mothers, are a vulnerable group with a challenging early parenting experience in caring for their infants. The findings of the study highlight some of the difficulties this population face whilst having to care, and specifically feed, their preterm infants during the hospitalisation period. This includes: high stress levels, difficulty with initiating and maintaining lactation, reduced feeding abilities of their infants, limited reciprocal interaction with their infants and being in an unfamiliar hospital environment for prolonged periods with limited support. The HCPs working with preterm mother–infant dyads should be aware of and sensitive towards mothers’ realities, and accommodate these realities in their treatment programme. Firstly, increased feeding support from HCPs is required, and especially increased access to feeding intervention provided by speech-language therapists and lactation consultants. Secondly, HCPs involved in neonatal care should seek effective methods to promote the prescribed feeding practices. These methods should aim to develop internal motivation in the members of this vulnerable group to follow the prescribed feeding practices, whilst still accommodating the hardships they encounter. This will allow HCPs to utilise the neonatal period optimally and to improve maternal wellbeing, mother–infant bonding and attachment, and the developmental outcomes of the infant.

## Appendix 1

### Discussion schedule

#### Introduction question: General experience

Tell me more about your experience of being the mother of a preterm infant?

#### Section 1: Prematurity

Your baby was born prematurely. What does this mean to you?

Prompt: reason/differences to term infants

#### Section 2: Caregiving

Tell me more about your experience of having and caring for a preterm infant?

Prompt: initially/currently/adjustments required

#### Section 3: Communication (Interaction)

Tell me more about your interaction (talking, playing, exchanging messages) with your infant?

Prompt: mother’s attempts (when and how)/infant’s response/infant’s attempts (when and how)/mother’s response/differences to term infants/challenges (makes it hard to interact)/facilitators (makes it easy to interact)

#### Section 4: Feeding

Tell me more about the experience of feeding your infant?

Prompt: method/experience: good, bad, easy, hard/differences to term baby

#### Section 5: Maternal role/responsibilities

You are the mother of this infant. What does this role mean to you?

Prompt: responsibilities (what must you do for the baby)/responsibilities that only you can fulfil

#### Section 6: Additional stakeholders

Tell me more about the other important people in this infant’s life?

Prompt: responsibilities towards infant/responsibilities towards mother

#### Section 7: Information needs

If you think about the past couple of months since your infant’s birth – tell me more about the type of support or information that would have made the process easier for you?

Prompt: format of information/agent/current information sources (where, who, how)
